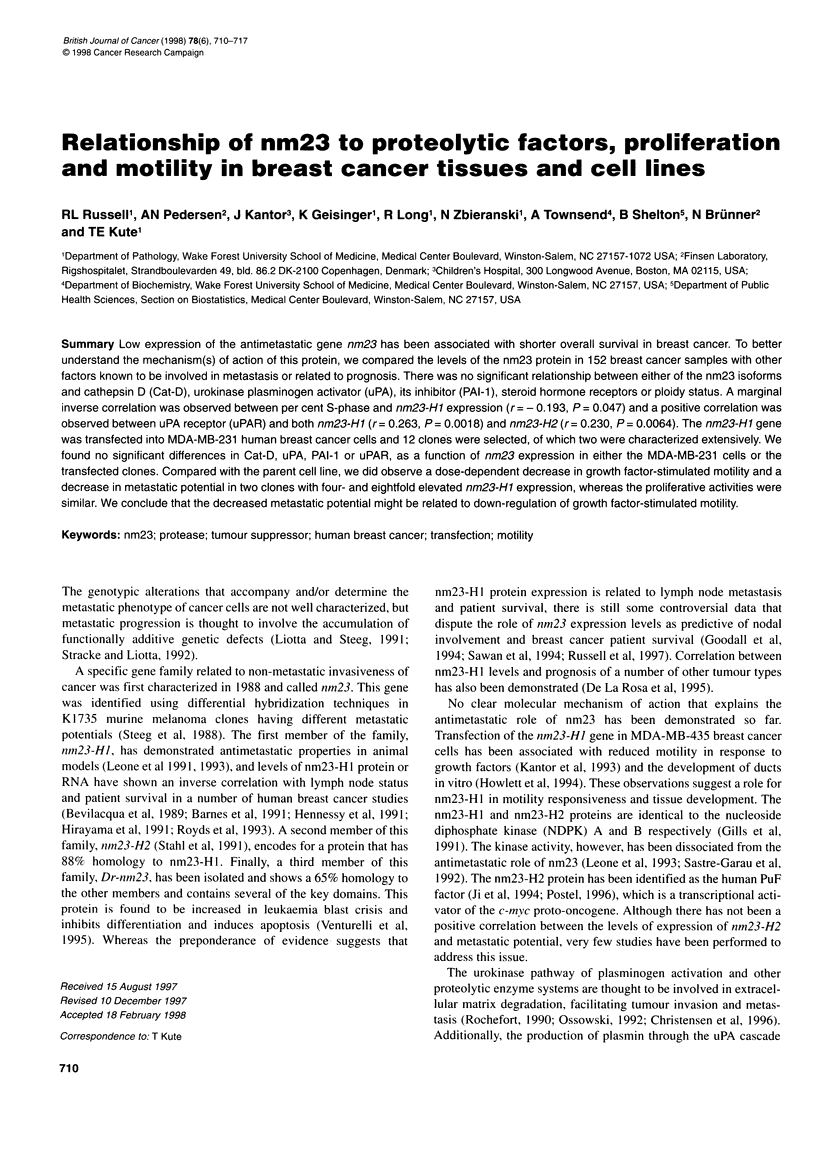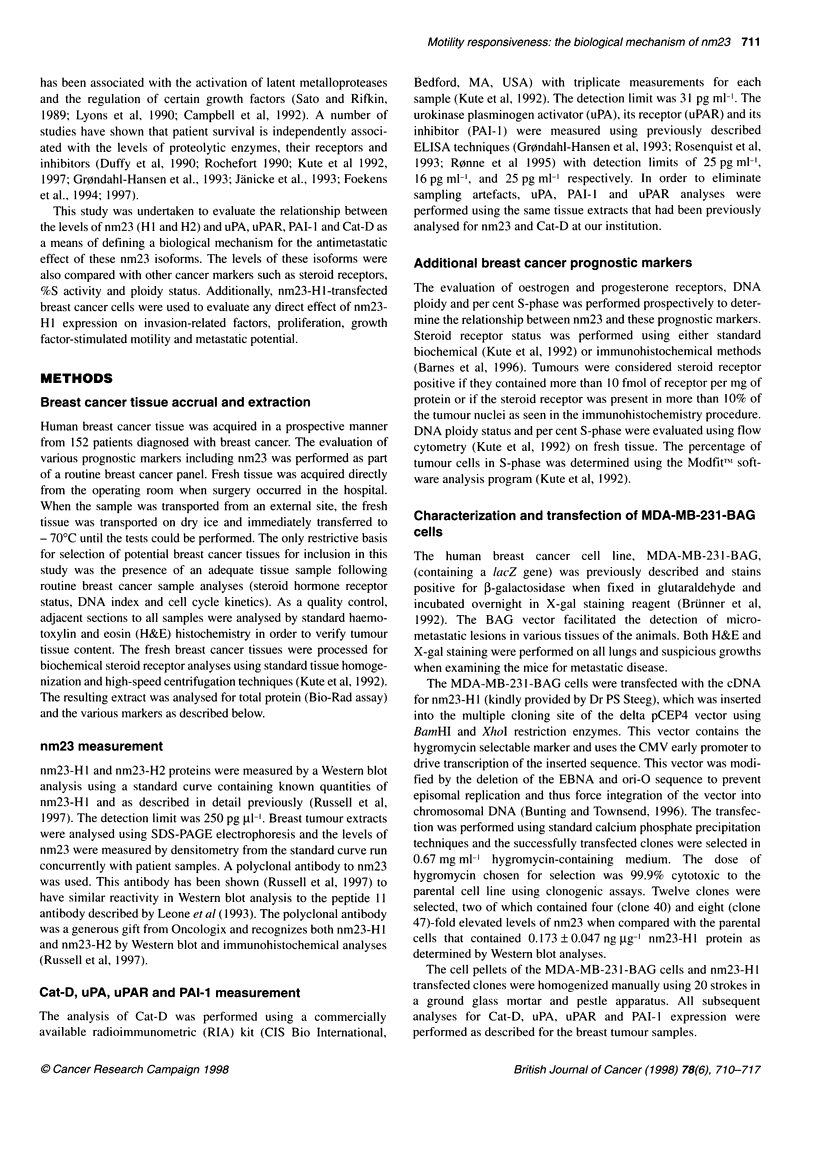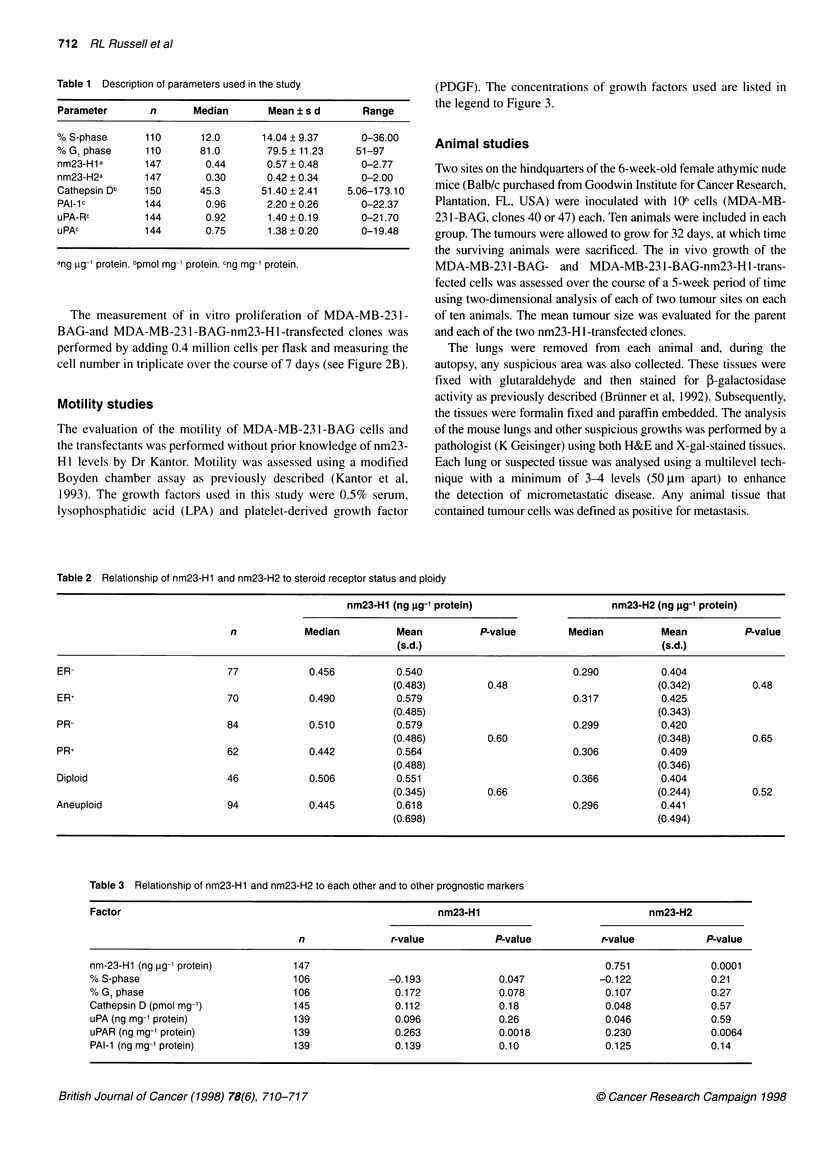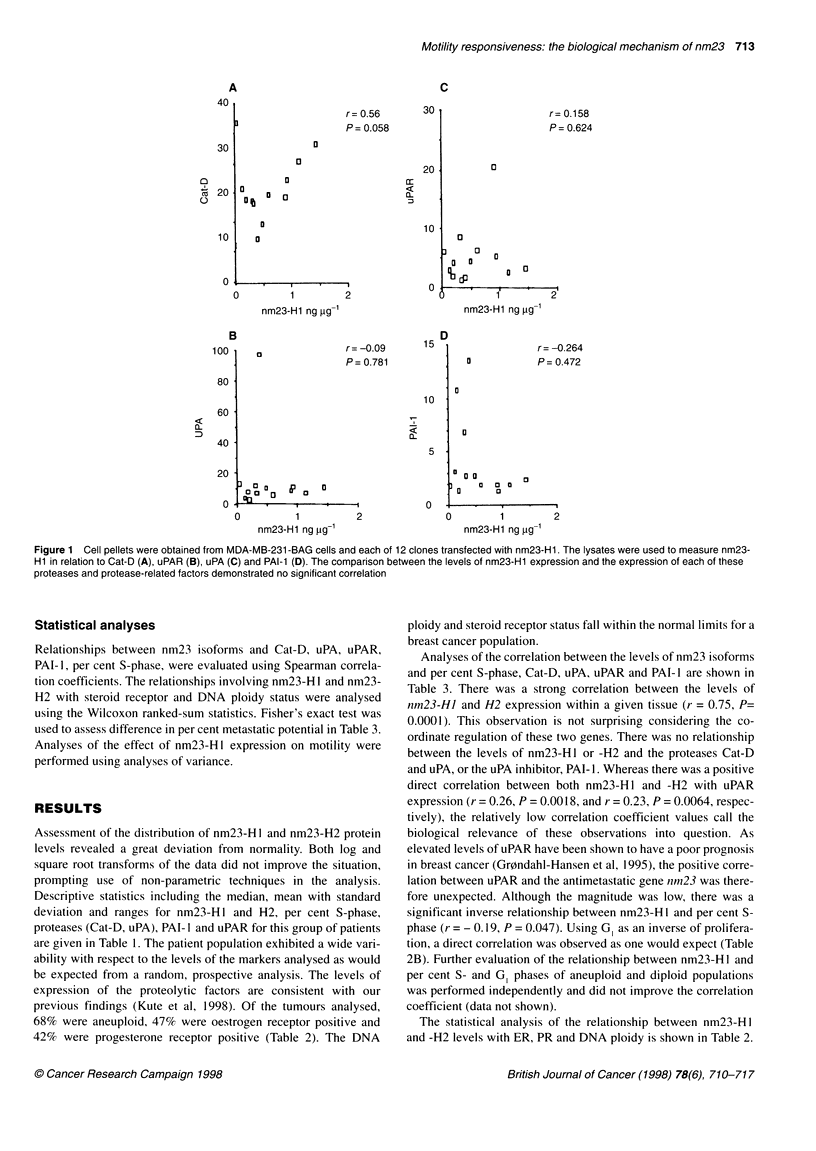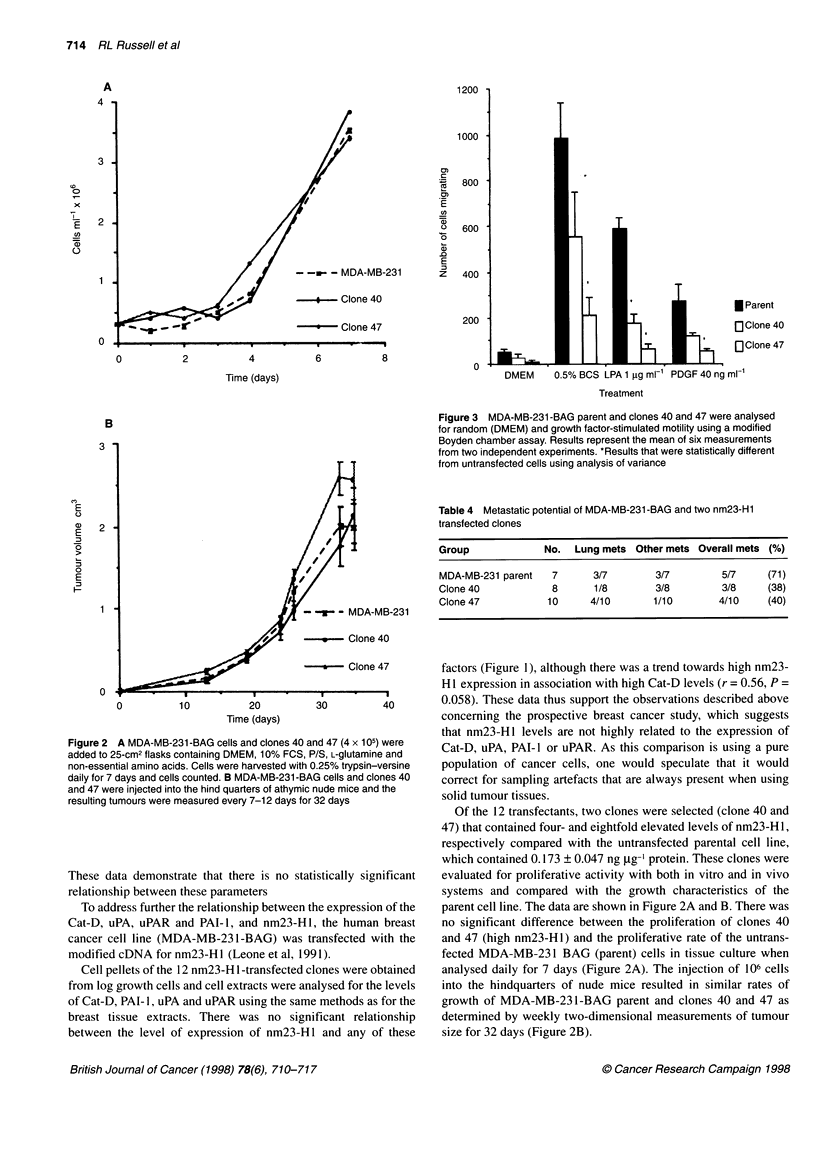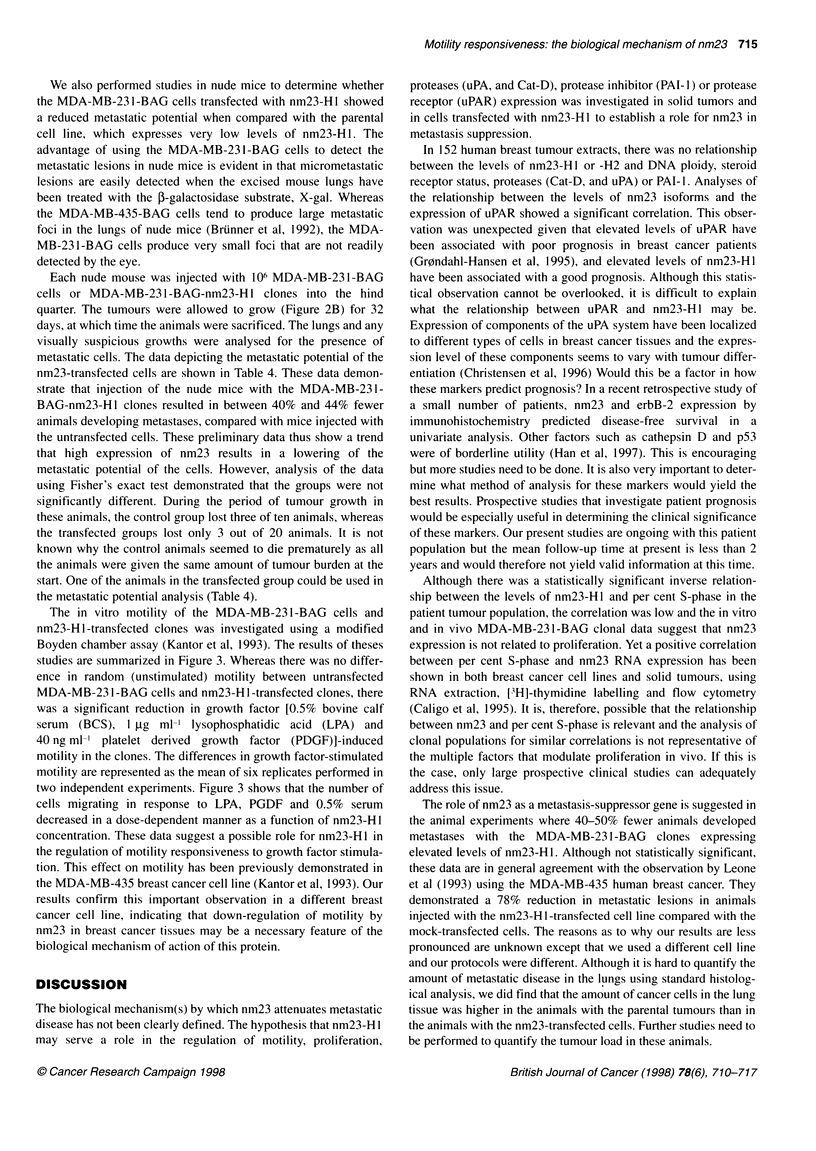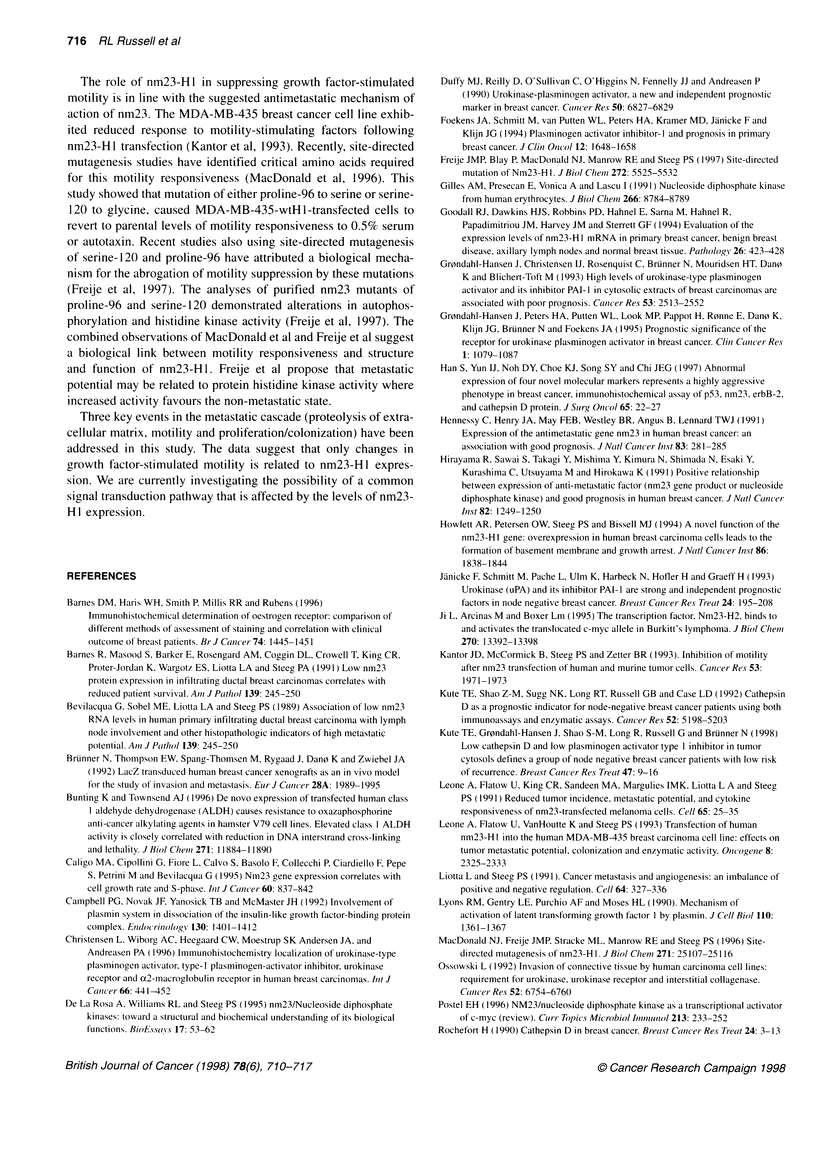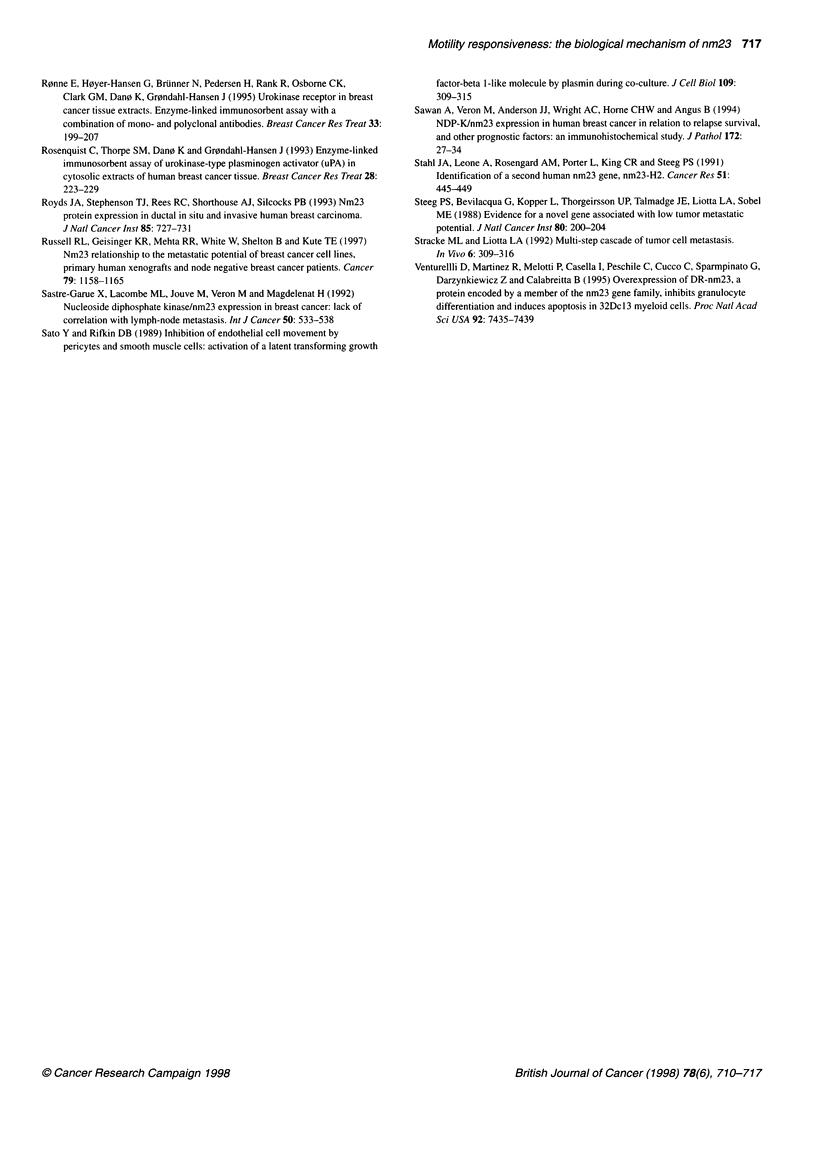# Relationship of nm23 to proteolytic factors, proliferation and motility in breast cancer tissues and cell lines.

**DOI:** 10.1038/bjc.1998.566

**Published:** 1998-09

**Authors:** R. L. Russell, A. N. Pedersen, J. Kantor, K. Geisinger, R. Long, N. Zbieranski, A. Townsend, B. Shelton, N. Brünner, T. E. Kute

**Affiliations:** Department of Pathology, Wake Forest University School of Medicine, Winston-Salem, NC 27157-1072, USA.

## Abstract

Low expression of the antimetastatic gene nm23 has been associated with shorter overall survival in breast cancer. To better understand the mechanism(s) of action of this protein, we compared the levels of the nm23 protein in 152 breast cancer samples with other factors known to be involved in metastasis or related to prognosis. There was no significant relationship between either of the nm23 isoforms and cathepsin D (Cat-D), urokinase plasminogen activator (uPA), its inhibitor (PAI-1), steroid hormone receptors or ploidy status. A marginal inverse correlation was observed between per cent S-phase and nm23-H1 expression (r = -0.193, P = 0.047) and a positive correlation was observed between uPA receptor (uPAR) and both nm23-H1 (r = 0.263, P = 0.0018) and nm23-H2 (r = 0.230, P = 0.0064). The nm23-H1 gene was transfected into MDA-MB-231 human breast cancer cells and 12 clones were selected, of which two were characterized extensively. We found no significant differences in Cat-D, uPA, PAI-1 or uPAR, as a function of nm23 expression in either the MDA-MB-231 cells or the transfected clones. Compared with the parent cell line, we did observe a dose-dependent decrease in growth factor-stimulated motility and a decrease in metastatic potential in two clones with four- and eightfold elevated nm23-H1 expression, whereas the proliferative activities were similar. We conclude that the decreased metastatic potential might be related to down-regulation of growth factor-stimulated motility.


British Journal of Cancer (1998) **78**(6), 710–717
© 1998 Cancer Research Campaign

### Relationship of nm23 to proteolytic factors, proliferation and motility in breast cancer tissues and cell lines

**RL Russell[1], AN Pedersen[2], J Kantor[3], K Geisinger[1], R Long[1], N Zbieranski[1], A Townsend[4], B Shelton[5], N Brünner[2] and TE Kute[1]**

[1]Department of Pathology, Wake Forest University School of Medicine, Medical Center Boulevard, Winston-Salem, NC 27157-1072 USA; [2]Finsen Laboratory, Rigshospitalet, Strandboulevarden 49, bld. 86.2 DK-2100 Copenhagen, Denmark; [3]Children's Hospital, 300 Longwood Avenue, Boston, MA 02115, USA; [4]Department of Biochemistry, Wake Forest University School of Medicine, Medical Center Boulevard, Winston-Salem, NC 27157, USA; [5]Department of Public Health Sciences, Section on Biostatistics, Medical Center Boulevard, Winston-Salem, NC 27157, USA

**Summary** Low expression of the antimetastatic gene *nm23* has been associated with shorter overall survival in breast cancer. To better understand the mechanism(s) of action of this protein, we compared the levels of the nm23 protein in 152 breast cancer samples with other factors known to be involved in metastasis or related to prognosis. There was no significant relationship between either of the nm23 isoforms and cathepsin D (Cat-D), urokinase plasminogen activator (uPA), its inhibitor (PAI-1), steroid hormone receptors or ploidy status. A marginal inverse correlation was observed between per cent S-phase and *nm23-H1* expression ($r = -0.193$, $P = 0.047$) and a positive correlation was observed between uPA receptor (uPAR) and both *nm23-H1* ($r = 0.263$, $P = 0.0018$) and *nm23-H2* ($r = 0.230$, $P = 0.0064$). The *nm23-H1* gene was transfected into MDA-MB-231 human breast cancer cells and 12 clones were selected, of which two were characterized extensively. We found no significant differences in Cat-D, uPA, PAI-1 or uPAR, as a function of *nm23* expression in either the MDA-MB-231 cells or the transfected clones. Compared with the parent cell line, we did observe a dose-dependent decrease in growth factor-stimulated motility and a decrease in metastatic potential in two clones with four- and eightfold elevated *nm23-H1* expression, whereas the proliferative activities were similar. We conclude that the decreased metastatic potential might be related to down-regulation of growth factor-stimulated motility.

**Keywords:** nm23; protease; tumour suppressor; human breast cancer; transfection; motility

The genotypic alterations that accompany and/or determine the metastatic phenotype of cancer cells are not well characterized, but metastatic progression is thought to involve the accumulation of functionally additive genetic defects (Liotta and Steeg, 1991; Stracke and Liotta, 1992).

A specific gene family related to non-metastatic invasiveness of cancer was first characterized in 1988 and called *nm23*. This gene was identified using differential hybridization techniques in K1735 murine melanoma clones having different metastatic potentials (Steeg et al, 1988). The first member of the family, *nm23-H1*, has demonstrated antimetastatic properties in animal models (Leone et al 1991, 1993), and levels of nm23-H1 protein or RNA have shown an inverse correlation with lymph node status and patient survival in a number of human breast cancer studies (Bevilacqua et al, 1989; Barnes et al, 1991; Hennessy et al, 1991; Hirayama et al, 1991; Royds et al, 1993). A second member of this family, *nm23-H2* (Stahl et al, 1991), encodes for a protein that has 88% homology to nm23-H1. Finally, a third member of this family, *Dr-nm23*, has been isolated and shows a 65% homology to the other members and contains several of the key domains. This protein is found to be increased in leukaemia blast crisis and inhibits differentiation and induces apoptosis (Venturelli et al, 1995). Whereas the preponderance of evidence suggests that nm23-H1 protein expression is related to lymph node metastasis and patient survival, there is still some controversial data that dispute the role of *nm23* expression levels as predictive of nodal involvement and breast cancer patient survival (Goodall et al, 1994; Sawan et al, 1994; Russell et al, 1997). Correlation between nm23-H1 levels and prognosis of a number of other tumour types has also been demonstrated (De La Rosa et al, 1995).

No clear molecular mechanism of action that explains the antimetastatic role of nm23 has been demonstrated so far. Transfection of the *nm23-H1* gene in MDA-MB-435 breast cancer cells has been associated with reduced motility in response to growth factors (Kantor et al, 1993) and the development of ducts in vitro (Howlett et al, 1994). These observations suggest a role for nm23-H1 in motility responsiveness and tissue development. The nm23-H1 and nm23-H2 proteins are identical to the nucleoside diphosphate kinase (NDPK) A and B respectively (Gills et al, 1991). The kinase activity, however, has been dissociated from the antimetastatic role of nm23 (Leone et al, 1993; Sastre-Garau et al, 1992). The nm23-H2 protein has been identified as the human PuF factor (Ji et al, 1994; Postel, 1996), which is a transcriptional activator of the *c-myc* proto-oncogene. Although there has not been a positive correlation between the levels of expression of *nm23-H2* and metastatic potential, very few studies have been performed to address this issue.

The urokinase pathway of plasminogen activation and other proteolytic enzyme systems are thought to be involved in extracellular matrix degradation, facilitating tumour invasion and metastasis (Rochefort, 1990; Ossowski, 1992; Christensen et al, 1996). Additionally, the production of plasmin through the uPA cascade

*Received 15 August 1997*
*Revised 10 December 1997*
*Accepted 18 February 1998*

*Correspondence to:* T Kute has been associated with the activation of latent metalloproteases and the regulation of certain growth factors (Sato and Rifkin, 1989; Lyons et al, 1990; Campbell et al, 1992). A number of studies have shown that patient survival is independently associated with the levels of proteolytic enzymes, their receptors and inhibitors (Duffy et al, 1990; Rochefort 1990; Kute et al 1992, 1997; Grøndahl-Hansen et al., 1993; Jänicke et al., 1993; Foekens et al., 1994; 1997).

This study was undertaken to evaluate the relationship between the levels of nm23 (H1 and H2) and uPA, uPAR, PAI-1 and Cat-D as a means of defining a biological mechanism for the antimetastatic effect of these nm23 isoforms. The levels of these isoforms were also compared with other cancer markers such as steroid receptors, %S activity and ploidy status. Additionally, nm23-H1-transfected breast cancer cells were used to evaluate any direct effect of nm23-H1 expression on invasion-related factors, proliferation, growth factor-stimulated motility and metastatic potential.

### METHODS

### Breast cancer tissue accrual and extraction

Human breast cancer tissue was acquired in a prospective manner from 152 patients diagnosed with breast cancer. The evaluation of various prognostic markers including nm23 was performed as part of a routine breast cancer panel. Fresh tissue was acquired directly from the operating room when surgery occurred in the hospital. When the sample was transported from an external site, the fresh tissue was transported on dry ice and immediately transferred to – 70°C until the tests could be performed. The only restrictive basis for selection of potential breast cancer tissues for inclusion in this study was the presence of an adequate tissue sample following routine breast cancer sample analyses (steroid hormone receptor status, DNA index and cell cycle kinetics). As a quality control, adjacent sections to all samples were analysed by standard haemotoxylin and eosin (H&E) histochemistry in order to verify tumour tissue content. The fresh breast cancer tissues were processed for biochemical steroid receptor analyses using standard tissue homogenization and high-speed centrifugation techniques (Kute et al, 1992). The resulting extract was analysed for total protein (Bio-Rad assay) and the various markers as described below.

### nm23 measurement nm23-H1 and nm23-H2 proteins were measured by a Western blot analysis using a standard curve containing known quantities of nm23-H1 and as described in detail previously (Russell et al, 1997). The detection limit was 250 pg $\mu l^{-1}$. Breast tumour extracts were analysed using SDS-PAGE electrophoresis and the levels of nm23 were measured by densitometry from the standard curve run concurrently with patient samples. A polyclonal antibody to nm23 was used. This antibody has been shown (Russell et al, 1997) to have similar reactivity in Western blot analysis to the peptide 11 antibody described by Leone *et al* (1993). The polyclonal antibody was a generous gift from Oncologix and recognizes both nm23-H1 and nm23-H2 by Western blot and immunohistochemical analyses (Russell et al, 1997).

### Cat-D, uPA, uPAR and PAI-1 measurement

The analysis of Cat-D was performed using a commercially available radioimmunometric (RIA) kit (CIS Bio International, Bedford, MA, USA) with triplicate measurements for each sample (Kute et al, 1992). The detection limit was 31 pg $ml^{-1}$. The urokinase plasminogen activator (uPA), its receptor (uPAR) and its inhibitor (PAI-1) were measured using previously described ELISA techniques (Grøndahl-Hansen et al, 1993; Rosenquist et al, 1993; Rønne et al 1995) with detection limits of 25 pg $ml^{-1}$, 16 pg $ml^{-1}$, and 25 pg $ml^{-1}$ respectively. In order to eliminate sampling artefacts, uPA, PAI-1 and uPAR analyses were performed using the same tissue extracts that had been previously analysed for nm23 and Cat-D at our institution.

### Additional breast cancer prognostic markers

The evaluation of oestrogen and progesterone receptors, DNA ploidy and per cent S-phase was performed prospectively to determine the relationship between nm23 and these prognostic markers. Steroid receptor status was performed using either standard biochemical (Kute et al, 1992) or immunohistochemical methods (Barnes et al, 1996). Tumours were considered steroid receptor positive if they contained more than 10 fmol of receptor per mg of protein or if the steroid receptor was present in more than 10% of the tumour nuclei as seen in the immunohistochemistry procedure. DNA ploidy status and per cent S-phase were evaluated using flow cytometry (Kute et al, 1992) on fresh tissue. The percentage of tumour cells in S-phase was determined using the Modfit™ software analysis program (Kute et al, 1992).

### Characterization and transfection of MDA-MB-231-BAG cells

The human breast cancer cell line, MDA-MB-231-BAG, (containing a *lacZ* gene) was previously described and stains positive for β-galactosidase when fixed in glutaraldehyde and incubated overnight in X-gal staining reagent (Brünner et al, 1992). The BAG vector facilitated the detection of micrometastatic lesions in various tissues of the animals. Both H&E and X-gal staining were performed on all lungs and suspicious growths when examining the mice for metastatic disease.

The MDA-MB-231-BAG cells were transfected with the cDNA for nm23-H1 (kindly provided by Dr PS Steeg), which was inserted into the multiple cloning site of the delta pCEP4 vector using *Bam*HI and *Xho*I restriction enzymes. This vector contains the hygromycin selectable marker and uses the CMV early promoter to drive transcription of the inserted sequence. This vector was modified by the deletion of the EBNA and ori-O sequence to prevent episomal replication and thus force integration of the vector into chromosomal DNA (Bunting and Townsend, 1996). The transfection was performed using standard calcium phosphate precipitation techniques and the successfully transfected clones were selected in 0.67 mg $ml^{-1}$ hygromycin-containing medium. The dose of hygromycin chosen for selection was 99.9% cytotoxic to the parental cell line using clonogenic assays. Twelve clones were selected, two of which contained four (clone 40) and eight (clone 47)-fold elevated levels of nm23 when compared with the parental cells that contained $0.173 \pm 0.047$ ng $\mu g^{-1}$ nm23-H1 protein as determined by Western blot analyses.

The cell pellets of the MDA-MB-231-BAG cells and nm23-H1 transfected clones were homogenized manually using 20 strokes in a ground glass mortar and pestle apparatus. All subsequent analyses for Cat-D, uPA, uPAR and PAI-1 expression were performed as described for the breast tumour samples.

© Cancer Research Campaign 1998

**Table 1** Description of parameters used in the study

| Parameter | n | Median | Mean ± s d | Range |
|---|---|---|---|---|
| % S-phase | 110 | 12.0 | 14.04 ± 9.37 | 0–36.00 |
| % $G_1$ phase | 110 | 81.0 | 79.5 ± 11.23 | 51–97 |
| nm23-H1[a] | 147 | 0.44 | 0.57 ± 0.48 | 0–2.77 |
| nm23-H2[a] | 147 | 0.30 | 0.42 ± 0.34 | 0–2.00 |
| Cathepsin D[b] | 150 | 45.3 | 51.40 ± 2.41 | 5.06–173.10 |
| PAI-1[c] | 144 | 0.96 | 2.20 ± 0.26 | 0–22.37 |
| uPA-R[c] | 144 | 0.92 | 1.40 ± 0.19 | 0–21.70 |
| uPA[c] | 144 | 0.75 | 1.38 ± 0.20 | 0–19.48 |

[a]ng μg$^{-1}$ protein. [b]pmol mg$^{-1}$ protein. [c]ng mg$^{-1}$ protein.

The measurement of in vitro proliferation of MDA-MB-231-BAG-and MDA-MB-231-BAG-nm23-H1-transfected clones was performed by adding 0.4 million cells per flask and measuring the cell number in triplicate over the course of 7 days (see Figure 2B).

### Motility studies

The evaluation of the motility of MDA-MB-231-BAG cells and the transfectants was performed without prior knowledge of nm23-H1 levels by Dr Kantor. Motility was assessed using a modified Boyden chamber assay as previously described (Kantor et al, 1993). The growth factors used in this study were 0.5% serum, lysophosphatidic acid (LPA) and platelet-derived growth factor (PDGF). The concentrations of growth factors used are listed in the legend to Figure 3.

### Animal studies

Two sites on the hindquarters of the 6-week-old female athymic nude mice (Balb/c purchased from Goodwin Institute for Cancer Research, Plantation, FL, USA) were inoculated with $10^6$ cells (MDA-MB-231-BAG, clones 40 or 47) each. Ten animals were included in each group. The tumours were allowed to grow for 32 days, at which time the surviving animals were sacrificed. The in vivo growth of the MDA-MB-231-BAG- and MDA-MB-231-BAG-nm23-H1-transfected cells was assessed over the course of a 5-week period of time using two-dimensional analysis of each of two tumour sites on each of ten animals. The mean tumour size was evaluated for the parent and each of the two nm23-H1-transfected clones.

The lungs were removed from each animal and, during the autopsy, any suspicious area was also collected. These tissues were fixed with glutaraldehyde and then stained for β-galactosidase activity as previously described (Brünner et al, 1992). Subsequently, the tissues were formalin fixed and paraffin embedded. The analysis of the mouse lungs and other suspicious growths was performed by a pathologist (K Geisinger) using both H&E and X-gal-stained tissues. Each lung or suspected tissue was analysed using a multilevel technique with a minimum of 3–4 levels (50 μm apart) to enhance the detection of micrometastatic disease. Any animal tissue that contained tumour cells was defined as positive for metastasis.

**Table 2** Relationship of nm23-H1 and nm23-H2 to steroid receptor status and ploidy

| | | nm23-H1 (ng μg$^{-1}$ protein) | | | nm23-H2 (ng μg$^{-1}$ protein) | | |
|---|---|---|---|---|---|---|---|
| | n | Median | Mean (s.d.) | P-value | Median | Mean (s.d.) | P-value |
| $ER^-$ | 77 | 0.456 | 0.540 (0.483) | 0.48 | 0.290 | 0.404 (0.342) | 0.48 |
| $ER^+$ | 70 | 0.490 | 0.579 (0.485) | | 0.317 | 0.425 (0.343) | |
| $PR^-$ | 84 | 0.510 | 0.579 (0.486) | 0.60 | 0.299 | 0.420 (0.348) | 0.65 |
| $PR^+$ | 62 | 0.442 | 0.564 (0.488) | | 0.306 | 0.409 (0.346) | |
| Diploid | 46 | 0.506 | 0.551 (0.345) | 0.66 | 0.366 | 0.404 (0.244) | 0.52 |
| Aneuploid | 94 | 0.445 | 0.618 (0.698) | | 0.296 | 0.441 (0.494) | |

**Table 3** Relationship of nm23-H1 and nm23-H2 to each other and to other prognostic markers

| Factor | | nm23-H1 | | nm23-H2 | |
|---|---|---|---|---|---|
| | n | r-value | P-value | r-value | P-value |
| nm-23-H1 (ng μg$^{-1}$ protein) | 147 | | | 0.751 | 0.0001 |
| % S-phase | 106 | −0.193 | 0.047 | −0.122 | 0.21 |
| % $G_1$ phase | 106 | 0.172 | 0.078 | 0.107 | 0.27 |
| Cathepsin D (pmol mg$^{-1}$) | 145 | 0.112 | 0.18 | 0.048 | 0.57 |
| uPA (ng mg$^{-1}$ protein) | 139 | 0.096 | 0.26 | 0.046 | 0.59 |
| uPAR (ng mg$^{-1}$ protein) | 139 | 0.263 | 0.0018 | 0.230 | 0.0064 |
| PAI-1 (ng mg$^{-1}$ protein) | 139 | 0.139 | 0.10 | 0.125 | 0.14 |

© Cancer Research Campaign 1998

**Figure 1** Cell pellets were obtained from MDA-MB-231-BAG cells and each of 12 clones transfected with nm23-H1. The lysates were used to measure nm23-H1 in relation to Cat-D (**A**), uPAR (**B**), uPA (**C**) and PAI-1 (**D**). The comparison between the levels of nm23-H1 expression and the expression of each of these proteases and protease-related factors demonstrated no significant correlation

### Statistical analyses

Relationships between nm23 isoforms and Cat-D, uPA, uPAR, PAI-1, per cent S-phase, were evaluated using Spearman correlation coefficients. The relationships involving nm23-H1 and nm23-H2 with steroid receptor and DNA ploidy status were analysed using the Wilcoxon ranked-sum statistics. Fisher's exact test was used to assess difference in per cent metastatic potential in Table 3. Analyses of the effect of nm23-H1 expression on motility were performed using analyses of variance.

### RESULTS

Assessment of the distribution of nm23-H1 and nm23-H2 protein levels revealed a great deviation from normality. Both log and square root transforms of the data did not improve the situation, prompting use of non-parametric techniques in the analysis. Descriptive statistics including the median, mean with standard deviation and ranges for nm23-H1 and H2, per cent S-phase, proteases (Cat-D, uPA), PAI-1 and uPAR for this group of patients are given in Table 1. The patient population exhibited a wide variability with respect to the levels of the markers analysed as would be expected from a random, prospective analysis. The levels of expression of the proteolytic factors are consistent with our previous findings (Kute et al, 1998). Of the tumours analysed, 68% were aneuploid, 47% were oestrogen receptor positive and 42% were progesterone receptor positive (Table 2). The DNA ploidy and steroid receptor status fall within the normal limits for a breast cancer population.

Analyses of the correlation between the levels of nm23 isoforms and per cent S-phase, Cat-D, uPA, uPAR and PAI-1 are shown in Table 3. There was a strong correlation between the levels of *nm23-H1* and *H2* expression within a given tissue ($r$ = 0.75, $P$= 0.0001). This observation is not surprising considering the coordinate regulation of these two genes. There was no relationship between the levels of nm23-H1 or -H2 and the proteases Cat-D and uPA, or the uPA inhibitor, PAI-1. Whereas there was a positive direct correlation between both nm23-H1 and -H2 with uPAR expression ($r$ = 0.26, $P$ = 0.0018, and $r$ = 0.23, $P$ = 0.0064, respectively), the relatively low correlation coefficient values call the biological relevance of these observations into question. As elevated levels of uPAR have been shown to have a poor prognosis in breast cancer (Grøndahl-Hansen et al, 1995), the positive correlation between uPAR and the antimetastatic gene *nm23* was therefore unexpected. Although the magnitude was low, there was a significant inverse relationship between nm23-H1 and per cent S-phase ($r$ = – 0.19, $P$ = 0.047). Using $G_1$ as an inverse of proliferation, a direct correlation was observed as one would expect (Table 2B). Further evaluation of the relationship between nm23-H1 and per cent S- and $G_1$ phases of aneuploid and diploid populations was performed independently and did not improve the correlation coefficient (data not shown).

The statistical analysis of the relationship between nm23-H1 and -H2 levels with ER, PR and DNA ploidy is shown in Table 2.

**Figure 2** **A** MDA-MB-231-BAG cells and clones 40 and 47 ($4 \times 10^5$) were added to 25-$cm^2$ flasks containing DMEM, 10% FCS, P/S, L-glutamine and non-essential amino acids. Cells were harvested with 0.25% trypsin–versine daily for 7 days and cells counted. **B** MDA-MB-231-BAG cells and clones 40 and 47 were injected into the hind quarters of athymic nude mice and the resulting tumours were measured every 7–12 days for 32 days

**Figure 3** MDA-MB-231-BAG parent and clones 40 and 47 were analysed for random (DMEM) and growth factor-stimulated motility using a modified Boyden chamber assay. Results represent the mean of six measurements from two independent experiments. *Results that were statistically different from untransfected cells using analysis of variance

**Table 4** Metastatic potential of MDA-MB-231-BAG and two nm23-H1 transfected clones

| Group | No. | Lung mets | Other mets | Overall mets | (%) |
|---|---|---|---|---|---|
| MDA-MB-231 parent | 7 | 3/7 | 3/7 | 5/7 | (71) |
| Clone 40 | 8 | 1/8 | 3/8 | 3/8 | (38) |
| Clone 47 | 10 | 4/10 | 1/10 | 4/10 | (40) |

These data demonstrate that there is no statistically significant relationship between these parameters

To address further the relationship between the expression of the Cat-D, uPA, uPAR and PAI-1, and nm23-H1, the human breast cancer cell line (MDA-MB-231-BAG) was transfected with the modified cDNA for nm23-H1 (Leone et al, 1991).

Cell pellets of the 12 nm23-H1-transfected clones were obtained from log growth cells and cell extracts were analysed for the levels of Cat-D, PAI-1, uPA and uPAR using the same methods as for the breast tissue extracts. There was no significant relationship between the level of expression of nm23-H1 and any of these factors (Figure 1), although there was a trend towards high nm23-H1 expression in association with high Cat-D levels ($r = 0.56$, $P = 0.058$). These data thus support the observations described above concerning the prospective breast cancer study, which suggests that nm23-H1 levels are not highly related to the expression of Cat-D, uPA, PAI-1 or uPAR. As this comparison is using a pure population of cancer cells, one would speculate that it would correct for sampling artefacts that are always present when using solid tumour tissues.

Of the 12 transfectants, two clones were selected (clone 40 and 47) that contained four- and eightfold elevated levels of nm23-H1, respectively compared with the untransfected parental cell line, which contained $0.173 \pm 0.047$ ng $\mu g^{-1}$ protein. These clones were evaluated for proliferative activity with both in vitro and in vivo systems and compared with the growth characteristics of the parent cell line. The data are shown in Figure 2A and B. There was no significant difference between the proliferation of clones 40 and 47 (high nm23-H1) and the proliferative rate of the untransfected MDA-MB-231 BAG (parent) cells in tissue culture when analysed daily for 7 days (Figure 2A). The injection of $10^6$ cells into the hindquarters of nude mice resulted in similar rates of growth of MDA-MB-231-BAG parent and clones 40 and 47 as determined by weekly two-dimensional measurements of tumour size for 32 days (Figure 2B).

 © Cancer Research Campaign 1998

We also performed studies in nude mice to determine whether the MDA-MB-231-BAG cells transfected with nm23-H1 showed a reduced metastatic potential when compared with the parental cell line, which expresses very low levels of nm23-H1. The advantage of using the MDA-MB-231-BAG cells to detect the metastatic lesions in nude mice is evident in that micrometastatic lesions are easily detected when the excised mouse lungs have been treated with the β-galactosidase substrate, X-gal. Whereas the MDA-MB-435-BAG cells tend to produce large metastatic foci in the lungs of nude mice (Brünner et al, 1992), the MDA-MB-231-BAG cells produce very small foci that are not readily detected by the eye.

Each nude mouse was injected with $10^6$ MDA-MB-231-BAG cells or MDA-MB-231-BAG-nm23-H1 clones into the hind quarter. The tumours were allowed to grow (Figure 2B) for 32 days, at which time the animals were sacrificed. The lungs and any visually suspicious growths were analysed for the presence of metastatic cells. The data depicting the metastatic potential of the nm23-transfected cells are shown in Table 4. These data demonstrate that injection of the nude mice with the MDA-MB-231-BAG-nm23-H1 clones resulted in between 40% and 44% fewer animals developing metastases, compared with mice injected with the untransfected cells. These preliminary data thus show a trend that high expression of nm23 results in a lowering of the metastatic potential of the cells. However, analysis of the data using Fisher's exact test demonstrated that the groups were not significantly different. During the period of tumour growth in these animals, the control group lost three of ten animals, whereas the transfected groups lost only 3 out of 20 animals. It is not known why the control animals seemed to die prematurely as all the animals were given the same amount of tumour burden at the start. One of the animals in the transfected group could be used in the metastatic potential analysis (Table 4).

The in vitro motility of the MDA-MB-231-BAG cells and nm23-H1-transfected clones was investigated using a modified Boyden chamber assay (Kantor et al, 1993). The results of theses studies are summarized in Figure 3. Whereas there was no difference in random (unstimulated) motility between untransfected MDA-MB-231-BAG cells and nm23-H1-transfected clones, there was a significant reduction in growth factor [0.5% bovine calf serum (BCS), 1 μg $ml^{-1}$ lysophosphatidic acid (LPA) and 40 ng $ml^{-1}$ platelet derived growth factor (PDGF)]-induced motility in the clones. The differences in growth factor-stimulated motility are represented as the mean of six replicates performed in two independent experiments. Figure 3 shows that the number of cells migrating in response to LPA, PGDF and 0.5% serum decreased in a dose-dependent manner as a function of nm23-H1 concentration. These data suggest a possible role for nm23-H1 in the regulation of motility responsiveness to growth factor stimulation. This effect on motility has been previously demonstrated in the MDA-MB-435 breast cancer cell line (Kantor et al, 1993). Our results confirm this important observation in a different breast cancer cell line, indicating that down-regulation of motility by nm23 in breast cancer tissues may be a necessary feature of the biological mechanism of action of this protein.

### DISCUSSION

The biological mechanism(s) by which nm23 attenuates metastatic disease has not been clearly defined. The hypothesis that nm23-H1 may serve a role in the regulation of motility, proliferation, proteases (uPA, and Cat-D), protease inhibitor (PAI-1) or protease receptor (uPAR) expression was investigated in solid tumors and in cells transfected with nm23-H1 to establish a role for nm23 in metastasis suppression.

In 152 human breast tumour extracts, there was no relationship between the levels of nm23-H1 or -H2 and DNA ploidy, steroid receptor status, proteases (Cat-D, and uPA) or PAI-1. Analyses of the relationship between the levels of nm23 isoforms and the expression of uPAR showed a significant correlation. This observation was unexpected given that elevated levels of uPAR have been associated with poor prognosis in breast cancer patients (Grøndahl-Hansen et al, 1995), and elevated levels of nm23-H1 have been associated with a good prognosis. Although this statistical observation cannot be overlooked, it is difficult to explain what the relationship between uPAR and nm23-H1 may be. Expression of components of the uPA system have been localized to different types of cells in breast cancer tissues and the expression level of these components seems to vary with tumour differentiation (Christensen et al, 1996) Would this be a factor in how these markers predict prognosis? In a recent retrospective study of a small number of patients, nm23 and erbB-2 expression by immunohistochemistry predicted disease-free survival in a univariate analysis. Other factors such as cathepsin D and p53 were of borderline utility (Han et al, 1997). This is encouraging but more studies need to be done. It is also very important to determine what method of analysis for these markers would yield the best results. Prospective studies that investigate patient prognosis would be especially useful in determining the clinical significance of these markers. Our present studies are ongoing with this patient population but the mean follow-up time at present is less than 2 years and would therefore not yield valid information at this time.

Although there was a statistically significant inverse relationship between the levels of nm23-H1 and per cent S-phase in the patient tumour population, the correlation was low and the in vitro and in vivo MDA-MB-231-BAG clonal data suggest that nm23 expression is not related to proliferation. Yet a positive correlation between per cent S-phase and nm23 RNA expression has been shown in both breast cancer cell lines and solid tumours, using RNA extraction, [$^3$H]-thymidine labelling and flow cytometry (Caligo et al, 1995). It is, therefore, possible that the relationship between nm23 and per cent S-phase is relevant and the analysis of clonal populations for similar correlations is not representative of the multiple factors that modulate proliferation in vivo. If this is the case, only large prospective clinical studies can adequately address this issue.

The role of nm23 as a metastasis-suppressor gene is suggested in the animal experiments where 40–50% fewer animals developed metastases with the MDA-MB-231-BAG clones expressing elevated levels of nm23-H1. Although not statistically significant, these data are in general agreement with the observation by Leone et al (1993) using the MDA-MB-435 human breast cancer. They demonstrated a 78% reduction in metastatic lesions in animals injected with the nm23-H1-transfected cell line compared with the mock-transfected cells. The reasons as to why our results are less pronounced are unknown except that we used a different cell line and our protocols were different. Although it is hard to quantify the amount of metastatic disease in the lungs using standard histological analysis, we did find that the amount of cancer cells in the lung tissue was higher in the animals with the parental tumours than in the animals with the nm23-transfected cells. Further studies need to be performed to quantify the tumour load in these animals.

© Cancer Research Campaign 1998 

The role of nm23-H1 in suppressing growth factor-stimulated motility is in line with the suggested antimetastatic mechanism of action of nm23. The MDA-MB-435 breast cancer cell line exhibited reduced response to motility-stimulating factors following nm23-H1 transfection (Kantor et al, 1993). Recently, site-directed mutagenesis studies have identified critical amino acids required for this motility responsiveness (MacDonald et al, 1996). This study showed that mutation of either proline-96 to serine or serine-120 to glycine, caused MDA-MB-435-wtH1-transfected cells to revert to parental levels of motility responsiveness to 0.5% serum or autotaxin. Recent studies also using site-directed mutagenesis of serine-120 and proline-96 have attributed a biological mechanism for the abrogation of motility suppression by these mutations (Freije et al, 1997). The analyses of purified nm23 mutants of proline-96 and serine-120 demonstrated alterations in autophosphorylation and histidine kinase activity (Freije et al, 1997). The combined observations of MacDonald et al and Freije et al suggest a biological link between motility responsiveness and structure and function of nm23-H1. Freije et al propose that metastatic potential may be related to protein histidine kinase activity where increased activity favours the non-metastatic state.

Three key events in the metastatic cascade (proteolysis of extracellular matrix, motility and proliferation/colonization) have been addressed in this study. The data suggest that only changes in growth factor-stimulated motility is related to nm23-H1 expression. We are currently investigating the possibility of a common signal transduction pathway that is affected by the levels of nm23-H1 expression.

### REFERENCES

Barnes DM, Haris WH, Smith P, Millis RR and Rubens (1996) Immunohistochemical determination of oestrogen receptor: comparison of different methods of assessment of staining and correlation with clinical outcome of breast patients. *Br J Cancer* **74**: 1445–1451

Barnes R, Masood S, Barker E, Rosengard AM, Coggin DL, Crowell T, King CR, Proter-Jordan K, Wargotz ES, Liotta LA and Steeg PA (1991) Low nm23 protein expression in infiltrating ductal breast carcinomas correlates with reduced patient survival. *Am J Pathol* **139**: 245–250

Bevilacqua G, Sobel ME, Liotta LA and Steeg PS (1989) Association of low nm23 RNA levels in human primary infiltrating ductal breast carcinoma with lymph node involvement and other histopathologic indicators of high metastatic potential. *Am J Pathol* **139**: 245–250

Brünner N, Thompson EW, Spang-Thomsen M, Rygaad J, Danø K and Zwiebel JA (1992) LacZ transduced human breast cancer xenografts as an in vivo model for the study of invasion and metastasis. *Eur J Cancer* **28A**: 1989–1995

Bunting K and Townsend AJ (1996) De novo expression of transfected human class 1 aldehyde dehydrogenase (ALDH) causes resistance to oxazaphosphorine anti-cancer alkylating agents in hamster V79 cell lines. Elevated class 1 ALDH activity is closely correlated with reduction in DNA interstrand cross-linking and lethality. *J Biol Chem* **271**: 11884–11890

Caligo MA, Cipollini G, Fiore L, Calvo S, Basolo F, Collecchi P, Ciardiello F, Pepe S, Petrini M and Bevilacqua G (1995) Nm23 gene expression correlates with cell growth rate and S-phase. *Int J Cancer* **60**: 837–842

Campbell PG, Novak JF, Yanosick TB and McMaster JH (1992) Involvement of plasmin system in dissociation of the insulin-like growth factor-binding protein complex. *Endocrinology* **130**: 1401–1412

Christensen L, Wiborg AC, Heegaard CW, Moestrup SK Andersen JA, and Andreasen PA (1996) Immunohistochemistry localization of urokinase-type plasminogen activator, type-1 plasminogen-activator inhibitor, urokinase receptor and α2-macroglobulin receptor in human breast carcinomas. *Int J Cancer* **66**: 441–452

De La Rosa A, Williams RL and Steeg PS (1995) nm23/Nucleoside diphosphate kinases: toward a structural and biochemical understanding of its biological functions. *BioEssays* **17**: 53–62

Duffy MJ, Reilly D, O'Sullivan C, O'Higgins N, Fennelly JJ and Andreasen P (1990) Urokinase-plasminogen activator, a new and independent prognostic marker in breast cancer. *Cancer Res* **50**: 6827–6829

Foekens JA, Schmitt M, van Putten WL, Peters HA, Kramer MD, Jänicke F and Klijn JG (1994) Plasminogen activator inhibitor-1 and prognosis in primary breast cancer. *J Clin Oncol* **12**: 1648–1658

Freije JMP, Blay P, MacDonald NJ, Manrow RE and Steeg PS (1997) Site-directed mutation of Nm23-H1. *J Biol Chem* **272**: 5525–5532

Gilles AM, Presecan E, Vonica A and Lascu I (1991) Nucleoside diphosphate kinase from human erythrocytes. *J Biol Chem* **266**: 8784–8789

Goodall RJ, Dawkins HJS, Robbins PD, Hahnel E, Sarna M, Hahnel R, Papadimitriou JM, Harvey JM and Sterrett GF (1994) Evaluation of the expression levels of nm23-H1 mRNA in primary breast cancer, benign breast disease, axillary lymph nodes and normal breast tissue. *Pathology* **26**: 423–428

Grøndahl-Hansen J, Christensen IJ, Rosenquist C, Brünner N, Mouridsen HT, Danø K and Blichert-Toft M (1993) High levels of urokinase-type plasminogen activator and its inhibitor PAI-1 in cytosolic extracts of breast carcinomas are associated with poor prognosis. *Cancer Res* **53**: 2513–2552

Grøndahl-Hansen J, Peters HA, Putten WL, Look MP, Pappot H, Rønne E, Danø K, Klijn JG, Brünner N and Foekens JA (1995) Prognostic significance of the receptor for urokinase plasminogen activator in breast cancer. *Clin Cancer Res* **1**: 1079–1087

Han S, Yun IJ, Noh DY, Choe KJ, Song SY and Chi JEG (1997) Abnormal expression of four novel molecular markers represents a highly aggressive phenotype in breast cancer, immunohistochemical assay of p53, nm23, erbB-2, and cathepsin D protein. *J Surg Oncol* **65**: 22–27

Hennessy C, Henry JA, May FEB, Westley BR, Angus B, Lennard TWJ (1991) Expression of the antimetastatic gene nm23 in human breast cancer: an association with good prognosis. *J Natl Cancer Inst* **83**: 281–285

Hirayama R, Sawai S, Takagi Y, Mishima Y, Kimura N, Shimada N, Esaki Y, Kurashima C, Utsuyama M and Hirokawa K (1991) Positive relationship between expression of anti-metastatic factor (nm23 gene product or nucleoside diphosphate kinase) and good prognosis in human breast cancer. *J Natl Cancer Inst* **82**: 1249–1250

Howlett AR, Petersen OW, Steeg PS and Bissell MJ (1994) A novel function of the nm23-H1 gene: overexpression in human breast carcinoma cells leads to the formation of basement membrane and growth arrest. *J Natl Cancer Inst* **86**: 1838–1844

Jänicke F, Schmitt M, Pache L, Ulm K, Harbeck N, Hofler H and Graeff H (1993) Urokinase (uPA) and its inhibitor PAI-1 are strong and independent prognostic factors in node negative breast cancer. *Breast Cancer Res Treat* **24**: 195–208

Ji L, Arcinas M and Boxer Lm (1995) The transcription factor, Nm23-H2, binds to and activates the translocated c-myc allele in Burkitt's lymphoma. *J Biol Chem* **270**: 13392–13398

Kantor JD, McCormick B, Steeg PS and Zetter BR (1993). Inhibition of motility after nm23 transfection of human and murine tumor cells. *Cancer Res* **53**: 1971–1973

Kute TE, Shao Z-M, Sugg NK, Long RT, Russell GB and Case LD (1992) Cathepsin D as a prognostic indicator for node-negative breast cancer patients using both immunoassays and enzymatic assays. *Cancer Res* **52**: 5198–5203

Kute TE, Grøndahl-Hansen J, Shao S-M, Long R, Russell G and Brünner N (1998) Low cathepsin D and low plasminogen activator type 1 inhibitor in tumor cytosols defines a group of node negative breast cancer patients with low risk of recurrence. *Breast Cancer Res Treat* **47**: 9–16

Leone A, Flatow U, King CR, Sandeen MA, Margulies IMK, Liotta L A and Steeg PS (1991) Reduced tumor incidence, metastatic potential, and cytokine responsiveness of nm23-transfected melanoma cells. *Cell* **65**: 25–35

Leone A, Flatow U, VanHoutte K and Steeg PS (1993) Transfection of human nm23-H1 into the human MDA-MB-435 breast carcinoma cell line: effects on tumor metastatic potential, colonization and enzymatic activity. *Oncogene* **8**: 2325–2333

Liotta L and Steeg PS (1991). Cancer metastasis and angiogenesis: an imbalance of positive and negative regulation. *Cell* **64**: 327–336

Lyons RM, Gentry LE, Purchio AF and Moses HL (1990). Mechanism of activation of latent transforming growth factor 1 by plasmin. *J Cell Biol* **110**: 1361–1367

MacDonald NJ, Freije JMP, Stracke ML, Manrow RE and Steeg PS (1996) Site-directed mutagenesis of nm23-H1. *J Biol Chem* **271**: 25107–25116

Ossowski L (1992) Invasion of connective tissue by human carcinoma cell lines: requirement for urokinase, urokinase receptor and interstitial collagenase. *Cancer Res* **52**: 6754–6760

Postel EH (1996) NM23/nucleoside diphosphate kinase as a transcriptional activator of c-myc (review). *Curr Topics Microbiol Immunol* **213**: 233–252

Rochefort H (1990) Cathepsin D in breast cancer. *Breast Cancer Res Treat* **24**: 3–13

© Cancer Research Campaign 1998

Rønne E, Høyer-Hansen G, Brünner N, Pedersen H, Rank R, Osborne CK, Clark GM, Danø K, Grøndahl-Hansen J (1995) Urokinase receptor in breast cancer tissue extracts. Enzyme-linked immunosorbent assay with a combination of mono- and polyclonal antibodies. *Breast Cancer Res Treat* **33**: 199–207

Rosenquist C, Thorpe SM, Danø K and Grøndahl-Hansen J (1993) Enzyme-linked immunosorbent assay of urokinase-type plasminogen activator (uPA) in cytosolic extracts of human breast cancer tissue. *Breast Cancer Res Treat* **28**: 223–229

Royds JA, Stephenson TJ, Rees RC, Shorthouse AJ, Silcocks PB (1993) Nm23 protein expression in ductal in situ and invasive human breast carcinoma. *J Natl Cancer Inst* **85**: 727–731

Russell RL, Geisinger KR, Mehta RR, White W, Shelton B and Kute TE (1997) Nm23 relationship to the metastatic potential of breast cancer cell lines, primary human xenografts and node negative breast cancer patients. *Cancer* **79**: 1158–1165

Sastre-Garue X, Lacombe ML, Jouve M, Veron M and Magdelenat H (1992) Nucleoside diphosphate kinase/nm23 expression in breast cancer: lack of correlation with lymph-node metastasis. *Int J Cancer* **50**: 533–538

Sato Y and Rifkin DB (1989) Inhibition of endothelial cell movement by pericytes and smooth muscle cells: activation of a latent transforming growth factor-beta 1-like molecule by plasmin during co-culture. *J Cell Biol* **109**: 309–315

Sawan A, Veron M, Anderson JJ, Wright AC, Horne CHW and Angus B (1994) NDP-K/nm23 expression in human breast cancer in relation to relapse survival, and other prognostic factors: an immunohistochemical study. *J Pathol* **172**: 27–34

Stahl JA, Leone A, Rosengard AM, Porter L, King CR and Steeg PS (1991) Identification of a second human nm23 gene, nm23-H2. *Cancer Res* **51**: 445–449

Steeg PS, Bevilacqua G, Kopper L, Thorgeirsson UP, Talmadge JE, Liotta LA, Sobel ME (1988) Evidence for a novel gene associated with low tumor metastatic potential. *J Natl Cancer Inst* **80**: 200–204

Stracke ML and Liotta LA (1992) Multi-step cascade of tumor cell metastasis. *In Vivo* **6**: 309–316

Venturellli D, Martinez R, Melotti P, Casella I, Peschile C, Cucco C, Sparmpinato G, Darzynkiewicz Z and Calabreitta B (1995) Overexpression of DR-nm23, a protein encoded by a member of the nm23 gene family, inhibits granulocyte differentiation and induces apoptosis in 32Dc13 myeloid cells. *Proc Natl Acad Sci USA* **92**: 7435–7439

© Cancer Research Campaign 1998